# Emotion Regulation in Adolescent Males with Attention-Deficit Hyperactivity Disorder: Testing the Effects of Comorbid Conduct Disorder

**DOI:** 10.3390/brainsci5030369

**Published:** 2015-09-07

**Authors:** Clare Northover, Anita Thapar, Kate Langley, Stephanie van Goozen

**Affiliations:** 1 School of Psychology, Cardiff University, Cardiff, CF10 3AT, UK; E-Mails: NorthoverC@cardiff.ac.uk (C.N.); LangleyK@cardiff.ac.uk (K.L.); 2 MRC Centre for Neuropsychiatric Genetics and Genomics, Cardiff University, Cardiff, CF24 4HQ, UK; E-Mail: Thapar@cardiff.ac.uk

**Keywords:** emotion regulation, ADHD, conduct disorder, ultimatum game, aggression, callous-unemotional traits

## Abstract

Although attention-deficit hyperactivity disorder (ADHD) has been linked to emotion dysregulation, few studies have experimentally investigated this whilst controlling for the effects of comorbid conduct disorder (CD). Economic decision-making games that assess how individuals respond to offers varying in fairness have been used to study emotion regulation. The present study compared adolescent boys with ADHD (*n* = 90), ADHD + CD (*n* = 94) and typical controls (*n* = 47) on the Ultimatum Game and examined the contribution of ADHD and CD symptom scores and callous and unemotional traits to acceptance levels of unfair offers. There were no significant differences in acceptance rates of fair and highly unfair offers between groups, and only boys with ADHD did not significantly differ from the controls. However, the subgroup of boys with ADHD and additional high levels of aggressive CD symptoms rejected significantly more ambiguous (*i.e.*, moderately unfair) offers than any other subgroup, suggesting impaired emotion regulation in those with ADHD and aggressive CD. Correlations within the CD group showed that the rejection rate to moderately unfair offers was predicted by aggressive CD symptom severity, but not callous and unemotional traits. These findings highlight the fact that ADHD is a heterogeneous condition from an emotion regulation point of view.

## 1. Introduction

It has long been recognized that children with attention deficit hyperactivity disorder (ADHD) have difficulty regulating their emotions. In particular, research has shown that children with this disorder exhibit greater emotional reactivity [1], higher levels of negative affect [2] and lower levels of emotional awareness [3]. Emotion regulation is defined as an individual’s ability to modify an emotional state so as to promote adaptive, goal-oriented behaviours [4]. Emotion dysregulation arises when these adaptive processes are impaired, leading to behaviour that defeats the individual’s interests [5]. Although prevalence rates of emotion dysregulation in ADHD are high [6], the clinical significance of these findings and how specific they are to ADHD remain unclear. It has not yet been established, for example, whether deficits in emotion regulation are evident in all children with ADHD or perhaps only in a subgroup of children with this disorder.

In the early conceptualisation of ADHD, emotion dysregulation was considered a cardinal symptom [7]. It was only with the introduction of the DSM-III [8] that emotion regulation became an associated feature rather than a diagnostic criterion. The current conceptualization of ADHD is made up of two age-inappropriate behavioural dimensions, these being inattention and hyperactivity-impulsivity (ADHD; DSM 5; [9]). However, many argue that emotion dysregulation should take more of a consideration in the assessment of ADHD due to its impact on psychological, physical and social outcomes [10,11]. Now, conceptual theories of emotion regulation and ADHD can generally be characterised by three separate models; emotion dysregulation as a core feature of ADHD, emotion dysregulation as a distinct, but correlated dimension to ADHD or the addition of emotion dysregulation and ADHD as a distinct entity [5].

Barkley [12] argues that emotion dysregulation is a core feature of ADHD and stems from executive functioning difficulties at the neurological level. Specifically, the inability to inhibit responses causes difficulties with selective attention, hyperactivity and impulsivity inherent in ADHD, as well as an impaired ability to inhibit strong emotional responses. However, emotion dysregulation is a dimensional trait that undercuts the traditional divide between internalizing and externalizing diagnoses, and it is not unique to ADHD [13]. Regulation of emotions is compromised in children with disruptive behavioural disorders (DBDs), like conduct disorder (CD) and oppositional defiant disorder (ODD), as well as mood disorders. A recent study by Factor *et al.* [14] suggests that ADHD alone is not sufficient for children to display significantly impaired emotional regulation, but it is only in the presence of a comorbid disorder that this pattern of deficiency begins to emerge.

Between 30% and 50% of children with ADHD meet the criteria for conduct disorder [15], and this subgroup shows greater ADHD symptom severity than those with ADHD alone and worse outcomes [16]. This group also appears to have higher familial and genetic loading for ADHD [17,18], especially those with aggressive CD symptoms [19]. However, previous research on emotion regulation in children with ADHD has often not considered the effects of comorbid CD [20,21,22]. It is difficult therefore to know whether it is the core features of ADHD that are linked to emotion dysregulation or whether the relationship is explained by associated CD.

Studies on emotion regulation have primarily used retrospective, self-report questionnaires. Experimental studies (e.g., [23,24]) have often used frustration eliciting tasks to assess emotion regulation; for example, by asking participants to hide their emotions from a confederate competitor. In this type of paradigm, however, the participants have no real motive to regulate their emotion apart from complying with the experimenter’s demands. Economic decision-making games, such as the Ultimatum Game (UG), provide another way of measuring emotion regulation by assessing effects on decision making [25,26,27,28]. These paradigms involve two players interacting to decide how to divide a sum of money. One player (the proposer) offers a portion of the money to the second player (the responder). The responder can either accept the offer (in which case, both players split the money as proposed) or reject the offer (in which case, both players get nothing). Traditional economic theories, which view decision-making as a rational, cognitive process (e.g., [29]) state that all offers, regardless of their fairness, should be accepted. Previous studies, however, have found that offers made to the responder that are comparatively small, and therefore deemed as unfair (20% of the total), have a 50% chance of being rejected by most individuals [25,30].

Most individuals experience a negative emotional response and increased arousal when receiving unfair offers [31], and a number of studies provide evidence that emotion regulation processes are a critical component in the UG. Negative emotions, such as anger and frustration, provoke participants to penalise their opponent rather than to make a utilitarian choice [32,33], and the rejection of unfair offers increases when feelings of sadness are induced [34]. The percentage of accepted unfair offers is influenced by the use of specific emotion regulation strategies, such as reappraisal [35,36], and when participants are asked to “stay calm”, they accept more unfair offers [37], suggesting that the ability to regulate negative emotions is necessary for the (rational) acceptance of unfair offers.

The rejection of unfair offers has been found to be associated with activity in neural substrates involved in negative emotions, such as the amygdala [38] and anterior insula [39]. Ventromedial prefrontal cortex (VMPC) damage is reliably associated with poorly-controlled emotional responses. In response to relatively minor provocation or frustration, patients with such damage are often irritable, angry, argumentative and even abusive [40,41,42,43], yet generally show shallow affect. Similarities have been observed between patients with VMPC damage and patients with psychopathy [26,44,45,46], and Koenigs *et al.* [28] found similarly high rejection rates to the UG in VMPC damage patients and prisoners with low-anxiety psychopathy. In a community sample, Viera [47] showed that the rejection rate of unfair offers was associated with VMPC activity in those with high psychopathy scores compared to those with low psychopathy scores; they interpreted this as reflecting an angry reaction to the frustration of not obtaining the desired outcome.

The results of studies in children and adults assessing emotion regulation suggest that age is an important factor. It is consistently found that adolescents reject more unfair offers than younger children and adults [48,49,50], suggesting that there is a U-shaped developmental trajectory. This is consistent with the conceptualization of a peak in emotional reactivity during adolescence [51]. However, until now no studies have examined emotion regulation using the UG in a clinical sample of adolescents with ADHD.

When assessing the contributory effects of comorbid externalizing disorders, it is important to consider the clinical and aetiological heterogeneity of disorders, such as CD and ODD [6]; not all children who engage in antisocial behaviour will display emotion regulation problems. Frick and Morris [52] argue deficits in emotion regulation are likely to underlie conduct problems that involve the angry and overt confrontation of others (e.g., fighting and assault), but are less likely to be associated with conduct problems that are not associated with confrontation or negative affect (e.g., stealing, vandalism). Burt and Donnellan [53] also argue that there are unique personality correlates of different forms of antisocial behaviour. They found that aggression was uniquely predicted by high stress reaction (e.g., easily upset, has unaccountable mood changes), but this was not related to non-aggressive rulebreaking behaviour. This suggests that adolescents who display high aggressive CD symptoms might have more difficulty regulating their negative emotions during the UG and reject more unfair offers.

Callous-unemotional traits (CU) are another potentially important source of heterogeneity when looking at externalizing disorders. Such personality traits identify those at greater risk for severe antisocial behaviour [54] and reduced responsiveness to treatment [55]. The importance of such traits has been acknowledged by including limited prosocial emotions as a specifier for CD in the fifth edition of the Diagnostic and Statistical Manual of Mental Disorders (American Psychiatric Association; [9,56]). CU traits are characterised by low prosocial emotions and behaviours, including shallow or blunted affect, lack of guilt or remorse, physiological under arousal and low empathy. Individuals who lack empathy and are not concerned about the emotions of others may be less likely to be driven by anger and the motivation to punish the proposer. This was supported in a non-clinical sample, which found that students scoring high on psychopathic traits rejected fewer unfair offers, interpreted as favouring self-interest [57]. However, this contradicts Koenigs *et al*.’s [28] study of psychopathic inmates and also contrasts the results of more recent studies, which found no differences in rejection rates between high and low psychopathy scorers in community adults [47] and adolescents [58] or between healthy and (high psychopathy scoring) incarcerated individuals [59].

Only a few studies have examined emotion regulation using the UG in adolescents [48,49,50,58], and we are not aware of any study that has done so in a clinical sample of adolescents with ADHD. This study compared the decision-making of those with ADHD against those with ADHD and CD; with respect to the latter group, we distinguished between those with low aggressive CD symptoms and high aggressive CD symptoms. Within these groups, we also looked at the effect of additional CU traits. We included a sample of typically-developing adolescent males for comparison.

## 2. Experimental Section

### 2.1. Sample

Participants were recruited from the Child and Adolescent Mental Health Services and Community Child Health Clinics in Wales. Children in the sample were of British Caucasian origin and met research criteria for a lifetime diagnosis of ADHD. Children with any known clinical or research diagnosis of schizophrenia, bipolar disorder, autistic spectrum disorder (ASD), Tourette’s syndrome or with an IQ < 70, epilepsy, brain damage or any other neurological or genetic disorder were excluded from the study. In total, 204 adolescent males with ADHD (mean age = 13.95 years, SD = 1.82; age range 10–17 years) took part in the present study. No participants were stimulant naive, but participants who were currently being prescribed stimulant medication were asked to come off their medication at least 24 h prior to testing.

Male control participants (NCs), aged 13–18 (mean age = 15.14 years; *N* = 47) were recruited from local comprehensive schools and youth centres from relatively deprived areas in Cardiff. The Youth Self Report (YSR; [60]) was used to screen for ADHD and CD. All NCs completed the Wechsler Abbreviated Scale of Intelligence (WASI; [61]) and the UG. Nobody had estimated IQ scores of <70.

Ethical approval was obtained from the Wales Multicentre Research Ethics Committee. Informed written consent was obtained for all accompanying parents and adolescents aged over 16 years. Written assent was obtained for younger adolescents.

### 2.2. Clinical Measures

Child psychopathology was assessed using the Development and Well Being Assessment (DAWBA) structured interview using both parents and children as informants [62]. Parents completed the ADHD and ODD/CD sections and children the ODD/CD section of the DAWBA. All interviews were administered by trained psychologists, supervised by an experienced clinician (AT). Symptom scores and diagnoses were generated from the DAWBA according to DSM-IV criteria (the DSM-V had not been published at the start of the study; [63]). CD symptoms were considered present if endorsed by either the parent or child. Given previous findings suggesting that DSM-IV defined aggressive CD items’ index CD heterogeneity [19,54], those with CD were further examined according to whether or not they had a high (>3) number of aggressive symptoms (as defined by DSM).

ODD has previously been viewed as being part of a CD diagnostic spectrum. It is characterised as a less severe form of CD, which is often a developmental precursor to CD [64]. However, ODD has since been shown to have some important diagnostic utility; in particular, the ability to predict risk for later emotional disorders after controlling for CD [65]. In the DSM-5, the exclusion criterion for CD has now been removed from the diagnostic criteria for ODD. ODD was therefore also assessed using the DAWBA to control for differences between groups.

CU traits were measured using the Youth Psychopathic traits Inventory (YPI; [66]). The CU subscale of the YPI contains 15 items, and each item is answered on a 4-point Likert scale (score range 15–60). The reliability and convergent validity of the YPI with other measures of CU traits has been established [67,68].

Parent-rated emotional symptoms were assessed using the Strengths and Difficulties Questionnaire (SDQ; [69]). This was also completed as part of the DAWBA. The five emotional items (worries, unhappy, afraid, clingy, somatic) were scored on a 3-point Likert scale and summed to obtain a total emotional symptom score (score range 0–10).

Cognitive ability was re-assessed on all current participants using the Wechsler Abbreviated Scale of Intelligence [61], 2-subset form (vocabulary and matrix reasoning). All of the participants came from community clinics, and none were stimulant naive. Participants who continued to take stimulant medication were asked to come off medication 24 h prior to testing.

### 2.3. The Ultimatum Game

In the Ultimatum Game [26], two players are given the opportunity to split a sum of money. One player (the proposer) proposes a way to split an amount of money with another player (the responder). These offers vary in fairness, and the participant must simply accept or reject the offers made to them. If the responder accepts the offer, both players are paid accordingly. If the offer is rejected, neither player is paid. The participants were instructed that the offers were real and had been made by previous participants of the same study. This was made more believable by asking participants to propose their offer (out of the options below), and after they had done so, their offer was stored in the database for use in future research [28]. In fact, the experimenter predetermined the offers, and photographs of the opponents/responders were taken from the Amsterdam Dynamic Facial Expression Set [70].

Offers made were either 5/5 (keep 5, give 5 points), 6/4 (keep 6, give 4 points), 7/3 (keep 7, give 3 points), 8/2 (keep 8, give 2 points) or 9/1 (keep 9, give 1). Participants were the responders in a series of 22 trials, in which they saw a photograph of a different person during each trial who made them an offer. In accordance with Koenigs and Tranel’s [26] paper, offers were generated in the following frequencies: two offers of 5/5 distribution, two offers of 6/4, six offers of 7/3, six offers of 8/2 distribution and six offers of 9/1 distribution. Sanfey *et al.* (2003) asked participants to rate what offers they considered to be fair, irrespective of whether they decided to reject or accept an offer. Of their participants, 58% considered any offer less than 5/5 as unfair, with the remaining 42% deeming anything less than 7/3 to be unfair. Therefore, although 100% of the participants deemed the offers of 8/2 and 9/1 unfair, participants were divided with respect to the fairness of the 6/4 and 7/3 offers. For these reasons, the offers were grouped into three groups: truly fair (5/5), very unfair (8/2 and 9/1) and moderately unfair (7/3 and 6/4).

### 2.4. Data Analyses

Two ADHD participants had incomplete DAWBA data, so they could not be included. Eight ADHD participants did not complete the UG due to noncompliance; these participants had significantly more aggressive CD symptoms (*p* < 0.05). Ten participants had an estimated IQ < 70 and were excluded. Therefore, 184 ADHD participants were included in the analysis, as well as 47 control participants referred to as normal controls (NC). The ADHD group consisted of those with ADHD only (ADHD; *n* = 90). Those with additional CD (ADHD + CD; *n* = 94) were split into those with low aggressive CD symptoms (ADHD + CD/LA; *n* = 64) and high aggressive CD symptoms (ADHD + CD/HA *n* = 30), based on whether they were below or above the mean (mean = 3.33 symptoms). Between-subject ANOVAs were used to test differences in offers and acceptance rates between groups. Offers were grouped into three offer types: completely fair (5/5), moderately fair (6/4 and 7/3) and highly unfair (8/2 and 9/1). Because the UG variables were not normally distributed, follow up Bonferroni pairwise comparisons were bootstrapped and reported with 95% confidence intervals. Non-parametric Kruskal-Wallis tests were also conducted and found the same results.Effect sizes are reported as partial eta squared (η^2^*_p_*; small ≥0.01, medium ≥0.06, large ≥0.14; [71]). Anomalies (±3 standard deviations away from the mean for each group) were removed and replaced with the mean of that variable. One-way ANOVAs were used to compare demographic and clinical variables between groups. Spearman’s correlations and regressions examined the effect of these clinical variables on the UG outcome variables. Analyses were carried out using SPSS 16.0 (SPSS Inc., Chicago, IL, USA).

## 3. Results

The demographic data for the two subgroups and the results of between-group analyses are presented in Table 1.

Table 1 shows that there was a significant difference in age due to the control group being older than the three clinical groups. There was also a significant difference in estimated IQ, with the ADHD group having a higher IQ score than the two CD groups. Therefore, when significant results were found, follow-up ANCOVA tests were performed in order to analyse the effect with age and IQ as covariates.

There was no difference between the three clinical groups in ADHD severity or the emotion subscale of the SDQ. There was also no significant difference between the CD groups for CU traits and ODD symptoms, but these groups were significantly higher than the ADHD-only group.

**Table 1:** Demographic characteristics of the groups.

	NC (*N* = 47)		ADHD (*N* = 90)		ADHD + LA/CD (*N* = 64)		ADHD + HA/CD (*N* = 30)			
	Mean	SD	Mean	SD	Mean	SD	Mean	SD	Sig	Post Hoc
Age	15.19	1.33	13.70	1.89	14.28	1.80	13.47	1.55	*p* < 0.001	NC > ADHD and ADHD + LA/CD and ADHD + HA/CD
IQ	88.00	9.46	93.41	10.33	88.92	9.18	86.57	8.70	*p* < 0.05	ADHD > ADHD + LA/CD and ADHD + HA/CD
ADHD score	N/A	N/A	11.78	4.95	13.03	4.29	13.50	4.02	ns	
Total CD score	N/A	N/A	0.98	0.14	4.45	0.16	8.00	0.24	*p* < 0.001	ADHD < ADHD + LA/CD < ADHD+HA/CD
Aggressive CD score	N/A	N/A	0.20	0.43	1.19	0.79	3.50	0.63	*p* < 0.001	ADHD < ADHD + LA/CD < ADHD + HA/CD
Non-Aggressive CD score	N/A	N/A	0.78	0.73	3.27	1.36	4.50	1.93	*p* < 0.001	ADHD < ADHD + LA/CD < ADHD+HA/CD
CU traits	N/A	N/A	16.56	6.15	19.28	7.19	22.28	5.32	*p* < 0.001	ADHD < ADHD + LA/CD and ADHD + HA/CD
ODD score	N/A	N/A	2.91	2.46	4.41	2.72	5.37	2.53	*p* < 0.001	ADHD < ADHD + LA/CD and ADHD + HA/CD
Emotional symptoms	N/A	N/A	4.78	2.75	4.88	2.47	5.43	3.12	ns	

Note: LA, low aggressive; CD, conduct disorder; HA, high aggressive; ODD, oppositional defiant disorder. All between-group analyses were done using one-way ANOVAs; ADHD score = number of ADHD symptoms; total CD score = total number of CD symptoms; aggressive CD score = number of aggressive CD symptoms; non-aggressive CD score = number of non-aggressive CD symptoms; CU traits = callous-unemotional traits subscale score; ODD score = number of ODD symptoms; emotional symptoms = strengths and difficulties emotional symptom subscale score; Sig = significance value.

### The Ultimatum Game

First, the original proposed offers were compared (see Table 2). A between-subject ANOVA found no significant difference between groups: *F*(3, 227) = 1.48, *p* = 0.22, η*_p_*^2^ = 0.02.

**Table 2:** Offers proposed.

	NC (*N* = 47)		ADHD (*N* = 90)		ADHD + CD/LA (*N* = 64)		ADHD + CD/HA (*N* = 30)	
	Mean	SD	Mean	SD	Mean	SD	Mean	SD
Proposed Offer	1.77	1.13	1.97	1.41	2.30	1.63	2.34	1.78

Note: Offers were scored accordingly; 5/5 = 1, 6/4 = 2, 7/3 = 3, 8/2 = 4, 9/1 = 5.

**Figure 1:**
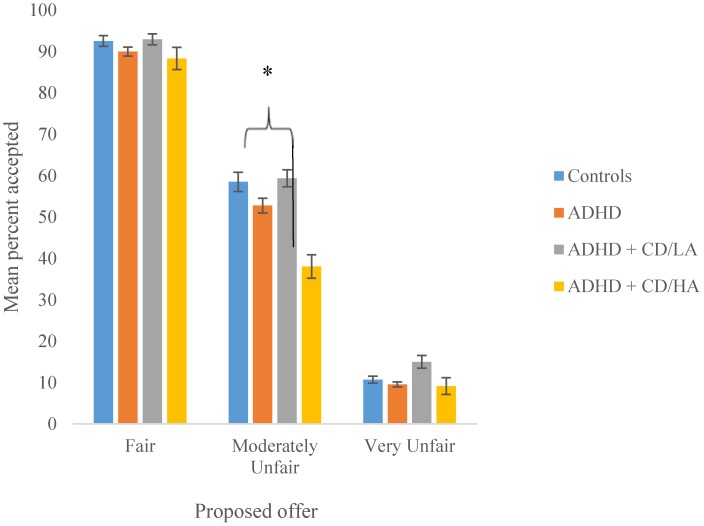
Percentage of Ultimatum Game offers accepted by adolescent males; normal controls, ADHD only, ADHD + CD/LA and ADHD + CD/HA. Error bars show ±1 SE. * *p* < 0.05.

We then compared the acceptance rates for the different offer types between the groups (see Figure 1). Between-group ANOVAs showed that the groups did not differ in acceptance rates for the fair offers (*F*(3, 227) = 0.53, *p* = 0.66, η*_p_*^2^ = 0.01) nor highly unfair offers (F(3, 227) = 2.36, *p* = 0.07, η*_p_*^2^ = 0.03). However, the groups did differ significantly in acceptance rates for the moderately unfair offers (*F*(3, 227) = 3.07, *p* = 0.03, *η_p_*^2^ = 0.04), and this remained significant after controlling for age and IQ (*p* = 0.03). The results of nonparametric Kruskal-Wallis tests confirmed these results: proposed offer, *χ*^2^ = 2.16, *p* = 0.54; fair offers, *χ*^2^ = 0.59, *p* = 0.90; moderately unfair offers, *χ*^2^ = 9.39, *p* = 0.03; highly unfair offers, *χ*^2^ = 2.43, *p* = 0.49. Follow-up bootstrapped pairwise comparisons showed that this effect was driven by the ADHD + CD/HA group rejecting significantly more offers than the other three groups (control group: *p* = 0.007, 95% CIs = −0.32, −0.06; ADHD only: *p* = 0.034, 95% CIs = −0.26, −0.01; ADHD + CD/LA: *p* = 0.005, 95% CIs = −0.34, −0.06).

Due to the difference found between the two CD groups, we wanted to explore the variation in symptoms between them. As shown in Table 1, the high aggressive group also reported more non-aggressive symptoms. It was therefore important to find out whether it was aggressive symptoms in particular or CD severity in general that predicted performance on the UG. Table 3 shows that both total CD score and aggressive CD score were significantly correlated with the acceptance rate of moderately unfair offers; none of the other demographic characteristics, including CU traits, were correlated.

**Table 3:** Correlations between the demographic characteristics and the acceptance rate of the moderately unfair offer of the adolescents with ADHD and comorbid CD.

	Age	IQ	ADHD	CD	Aggressive CD	CU Traits	ODD	Emotional Symptoms	Mod unfair
Age	-	-	-	-	-	-	-	-	-
IQ	0.02	-	-	-	-	-	-	-	-
ADHD	−0.21 *	0.24 *	-	-	-	-	-	-	-
CD	−0.14	−0.08	0.18	-	-	-	-	-	-
Aggressive CD	−0.32 **	−0.17	0.10	0.66 **	-	-	-	-	-
CU traits	−0.02	−0.05	0.12	0.21 *	0.16	-	-	-	-
ODD	−0.09	0.05	0.45 **	0.27 *	0.33 **	0.15	-	-	-
Emotional symptoms	−0.22 *	0.07	0.18	0.15	0.11	−0.11	0.16	-	-
Mod unfair	0.19	−0.05	−0.18	−0.21*	−0.25*	−0.11	−0.06	0.02	-

Note: ADHD = number of ADHD symptoms; CD = total number of CD symptoms; aggressive CD = number of aggressive CD symptoms; ODD = number of ODD symptoms; CU traits = callous-unemotional traits subscale score; emotional symptoms = strengths and difficulties emotional symptom subscale score; Mod unfair= acceptance rate of moderately unfair offers. * *p* < 0.05, ** *p* < 0.001.

A stepwise multiple regression was then conducted to evaluate whether both aggressive CD and total CD scores predicted the acceptance of moderately unfair offers. At Step 1 of the analysis, the aggressive CD score entered into the regression equation and was significantly negatively related to acceptance rates: *F*(1,92) = 5.11, *p* = 0.026, *R* = 0.23. Total CD score did not enter into the equation at Step 2 of the analysis (*t* = −0.246, *p* = 0.81).

## 4. Discussion

This study sought to examine whether ADHD adolescents in general have a problem with emotion regulation or whether this is a specific problem in those with conduct disorder, especially those with predominantly aggressive symptoms. No study until now has examined economic decision-making using the UG in a clinical sample of youths with ADHD, with or without CD. This study supports previous work [14] suggesting that children with ADHD show significantly higher levels of emotional dysregulation than control children only in the presence of a comorbid disorder.

Unsurprisingly, the vast majority of the adolescents accepted the fair offers and rejected the unfair offers. Generally our adolescent male groups accepted fewer unfair offers than those reported for adults [26,39]. This supports the suggestion of a peak in emotional reactivity during adolescence [51]. There were no differences between the four adolescent groups’ acceptance rates for fair (5/5) and seriously unfair offers (9/1 and 8/2); however, a significant group effect was found for the moderately unfair offers (6/4 and 7/3), suggesting that problems in emotion regulation become more apparent under ambiguous conditions.

Follow-up tests showed that the ADHD with aggressive CD group rejected significantly more moderately unfair offers than any other group. Previous studies claim that the rejection of unfair offers is due to anger and a desire to punish the opponent, and the responder’s ability to regulate anger and frustration therefore plays a critical role in task performance [32]. All three clinical groups reported the same amount of internalising emotionality in the SDQ. However, when faced with being treated unfairly, group differences in the ability to regulate externalising emotions became clear. Our results suggest that emotion regulation difficulties are not found in adolescents with ADHD alone, but rather only in those who have additional aggressive behaviour. This reflects their clinical presentation: being unable to control their aggressive behaviour [52].

The results suggest that ADHD alone is not associated with emotion dysregulation during the UG compared to normal adolescents, supporting the view that emotion dysregulation is not a core feature of ADHD. The fact that the biggest difference between groups was between the two CD groups highlights the importance of treating CD as a heterogeneous disorder. The results showed that aggressive symptoms predicted performance on the UG better than overall CD severity, supporting the idea that aggressive antisocial behaviour has a different aetiology than non-aggressive behaviour [19,54].

In the present study, participants were told that the aim of the game was to gain as many points as possible. Apart from this, there was no other incentive for them to win. The use of real reward incentives might, therefore, have a large impact on the rate of offers accepted. Further research is needed to help determine this in order to facilitate the development of more specific interventions for CD. For example, intervention programmes may be more beneficial by focusing on emotion regulation management in individuals with aggressive CD, whilst working with incentive-based goals in individuals with low-aggressive CD.

Unlike aggression, CU traits did not influence the acceptance of offers, supporting previous studies, which found no significant difference between individuals scoring high or low in psychopathy [47,58,59], but not others [28,57]. Previous studies have found that aggressive behaviour is positively correlated with negative emotionality and dysfunction [53], whereas CU traits are negatively correlated with these same traits [72]. In a modified version of the UG, Radke *et al.* [59] found that offenders high in psychopathy, like controls, took the context of the offer into consideration (*i.e.*, whether the proposer had a fair or unfair alternative offer to choose from), whereas offenders low in psychopathy did not, suggesting stronger impairments in social decision-making. However, even if similar behaviour patterns are shown in high and low psychopathy scorers, these might represent different motivations, as suggested by recent imaging studies [47,58], and this now needs to be tested further in clinical samples. Koenigs *et al.* [28] observed poorer regulation during the UG in low-anxious psychopathic offenders in comparison to high-anxious psychopathic and non-psychopathic offenders. However, due to the small sample sizes (*n* = 6) and lack of a non-ASB control sample, further research is needed. Our groups did not differ significantly in internalising emotionality (measured by the SDQ), and it may be the case that the low anxiety component of psychopathy drives regulation problems. Further research is needed using a clinical sample of children with disruptive behaviour problems, and improvements could be made by using a combination of parent, teacher and behavioural observation to assess CU traits, as suggested by the new CU specifier for the CD diagnosis in the DSM-5.

An issue that needs further exploration is the assumption that the acceptance of unfair offers is the rational decision. From an economic perspective, the rejection of offers is irrational, because it results in a personal loss. However, from a social perspective, rejection of unfair offers can be seen as a rational, altruistic action to preserve social norms. Rather than maximizing self-interest, the participant chooses to punish the socially-inappropriate action from the proposer for the good of the general population [73,74]. This would explain why similar rejection rates are found in a modified version of the UG in which the participants play on behalf of a third party, compared to one played by themselves [75]. We would argue that it is unlikely that boys high in aggressive CD symptoms rejected offers for the “good of the general population”, and this is supported by a recent imaging study, which found differences in response to the UG between severely antisocial adolescents and controls [76]. That study found decreased right inferior frontal gyrus (rIFG) activity in antisocial youngsters during the UG and no correlation between rIFG activity and behavioural responses, as was found in the controls. These results complement previous studies that suggest that juvenile antisocial behaviour is associated with difficulties in engaging the regulatory processes associated with the frontal cortex [77,78], in particular the rIFG, which is associated with response inhibition [79,80]. This supports the notion that the rejection of unfair offers in antisocial populations is due to deficient self-regulatory processes. Further research should now investigate more thoroughly participants’ reasoning behind the rejection of offers in order to support this.

Another limitation of the study is that during the UG task, no direct measure of participants’ emotional responses, such as psychophysiological recordings or subjective ratings, were obtained. Because we did not measure participants’ emotional response to offers, we do not know how much participants needed to self-regulate. One would assume that the more intense the shift in emotion, the more regulatory resources would be needed in order to modify that emotion. It is difficult, however, to determine from this study whether the boys with aggressive CD had deficient regulatory resources or experienced a more intense emotional reaction. Previous findings of reduced psychophysiological responding to aversive stimuli in the sample group would suggest the former [81,82]. Future research including such additional measures should help untangle the various potential factors affecting performance on the UG. The simplicity of the paradigm is also well suited for testing this sample using brain imaging techniques.

Emotion dysregulation is a dimensional trait that is not unique to ADHD. It is important to uncover to what extent individuals with ADHD and comorbid CD develop emotion regulation deficits for reasons that are different from those with CD alone by testing psychopathology in non-ADHD samples. Longitudinal studies are needed in order to define how the developmental trajectories interact with one another, to see, for example, whether emotion regulation difficulties bridge the development of aggressive behaviour in children and adolescents with ADHD or whether factors underlying both ADHD and comorbid aggressive CD (*i.e.*, temperamental or biological factors) lead children to demonstrate impairing levels of emotional dysregulation. Furthermore, existing treatments need to be modified to address the role of emotional regulation in children with ADHD. They should incorporate cognitive-behavioural techniques to teach emotion recognition and physiological relaxation exercises for negative emotions [83,84] and encourage problem-solving techniques to help children adjust and self-regulate when their expectations are not met [85].

## 5. Conclusions

In conclusion, this study of adolescent boys with ADHD and controls found a significant difference in the acceptance rate of ambiguously unfair offers of boys with ADHD and highly aggressive CD. The results suggest that it is the subgroup of boys with both ADHD and predominantly aggressive CD that has difficulty with emotion regulation, which causes them to make more “irrational” decisions. Importantly, boys with ADHD alone did not differ from controls in performance on the decision-making task. Further research is needed to better understand how emotion regulation influences decision-making and antisocial behaviour in the short and long term amongst adolescents, including those with psychiatric disorder.
